# The Diagnosis of Tuberculosis, Dealing Especially with the Dose of Tuberculin to Be Used in Testing for Tuberculosis*A paper read before the Maine Veterinary Medical Association.

**Published:** 1894-12

**Authors:** F. L. Russell

**Affiliations:** Professor of Veterinary Science, Maine State Agricultural College, and Experiment Station, Orono, Me.


### THE DIAGNOSIS OF TUBERCULOSIS, DEALING ESPECIALLY WITH THE DOSE OF TUBERCULIN TO BE USED IN TESTING FOR TUBERCULOSIS.*

By F. L. Russell, V.S.,
Professor of Veterinary Science, Maine State Agricultural College, and Experiment Station, Orono, Me.

We find that most of those who have published their experience in the use of tuberculin, have used a much larger dose of the drug than we have found necessary, and we wish to bring the matter briefly to your attention in a comparison of results, as it is a matter of general interest and some importance.

In this country the available tuberculin is mostly from one of two sources. It is either that prepared in Germany by Dr. Libbertz, after Prof. Koch's formula, or that made in this country by the Bureau of Animal Industry, at Washington, D. C. This latter preparation is a dilute tuberculin of about one-tenth the strength of the imported drug. Whether the strength of either brand is uniform, depends upon the care and skill exercised in their manufacture, and we will assume that all reasonable care is used, as we have no convenient method of testing them. But we find that from .2 to .5cc. of tuberculin, for mature animals, is generally recommended, while our own experience with a dose of .05cc., or even .06cc., have been very satisfactory.

In the New Jersey State College herd, the first cow tested, and one tested at a later date, were given .5cc. of tuberculin each, thirty-five cows were given 15cc. of tuberculin, and three animals were left untested for want of material. In one case reported in Pennsylvania .25cc. was used, with negative results, although an autopsy proved the cow to be tuberculous, and the failure was attributed to the smallness of the dose.

The point I wish to make is that the large dose, quite generally used, has no advantage over a much smaller one, but, on the contrary, some evident disadvantages. If thirty or forty animals, as they will be found in a breeding herd of all ages, can be tested with 1cc. of tuberculin, with as good results as when the same amount is used for four or five, the matter of expense alone is worth con-

* A paper read before the Maine Veterinary Medical Association.

sidering. It is well also to bear in mind that the larger the dose the greater the possibility that well animals will react.

In June, 1892, when we first used tuberculin, we were uncertain about the dose proper, but finally decided to try .05cc., and tested a herd of five mature cows, of rather more than average size, giving that dose. To our great surprise, where we had only suspected one, four reacted decidedly to the test. This unexpected result led us to believe that we might have used too large a dose. So, before killing these animals we tested another herd of twelve cows, dividing them into two lots of six each. One lot we injected with .05cc. tuberculin to a cow, and the other lot with .025cc. Five of these twelve cows gave decided reaction, and the diagnosis in each case was verified by the autopsy. From the smaller dose the highest temperature obtained were 105.5, 105.1, in fourteen hours from the time of injection. From the larger dose 106.7, 103.8, 105.5 in fourteen, sixteen and eighteen hours. This trial tended to confirm our opinion that .05cc. of tuberculin is at least sufficient to test a mature animal for tuberculosis, and in subsequent tests we have not exceeded that amount, but have used from .05cc. to .015cc., according to the age and size of the animal tested.

In all we have had thirty-one animals react under the test. Autopsies were made in each case, and the diagnosis was uncertain in but two instances. Most of the animals were but slightly affected. Five or six of them would be considered advanced cases, and were readily diagnosed by a physical examination. Two of the thirty-one were calves that were injected with .015cc. of tuberculin each. The thirty-one animals together were injected with less than 1.4cc of tuberculin.

Now to make a general comparison of results with some of those who have used a much larger dose.

We find that the average maximum temperature in these thirty-one cases was 105.4, and the average number of hours after injection before the maximum temperature was reached was fifteen hours.

In the Wisconsin Experiment Station herd from .4cc. to .1cc. of tuberculin to an animal was used.

The average highest temperature of the twenty-one that reacted under the first test was 105.5, and the average time to the highest temperature was fifteen and a half hours.

The New Jersey State College herd was tested with an average dose for mature animals of .31cc.

The average highest temperature of twenty-five animals that reacted was 104.9, in an average time of sixteen hours. One cow, in an advanced stage of tuberculosis, was given .5cc., but failed to react.

In December, 1893, the Vermont Experiment Station herd was tested with from .2cc. to .3cc. to each mature animal.

Twenty-three animals reacted with an average highest temperature of 105.8 in sixteen hours.

I might go on adding to these figures the record of other herds, but perhaps this is sufficient to show that the reaction is as decisive and as quickly obtained with a small as with a large dose of tuberculin.

